# Activities of daily living limitations and the use of physical examination among older adults with informal care in China: do gender and residence make differences?

**DOI:** 10.1186/s12877-024-04673-3

**Published:** 2024-01-23

**Authors:** Jingjing Luo, Dan Zhao, Tingting Gao, Jingjie Sun, Peilong Li, Xuehong Wang, Xueqing Wang, Shujun Chai, Jiayan Li, Chengchao Zhou

**Affiliations:** 1 Centre for Health Management and Policy Research, School of Public Health, Cheeloo College of Medicine, Shandong University, 44 Wen-hua-xi Road, 250012 Jinan, Shandong China; 2 Shandong Health Commission Medical Management Service Center, 250012 Jinan, China; 3 NHC Key Lab of Health Economics and Policy Research, Shandong University, 250012 Jinan, China

**Keywords:** ADL limitations, Physical examination, Gender disparity, Residence

## Abstract

**Background:**

This study investigated the relationship between activities of daily living (ADL) limitations and the use of physical examination among older adults receiving informal care, and to further examine whether this relationship varies by gender and urban-rural areas.

**Methods:**

The data in this study were obtained from the sixth Health Service of Shandong province, China. In total, 8,358 older adults aged 60 years or older who received informal care were included in the analysis. Binary logistic regression models were conducted to explore the association between ADL limitations and the use of physical examination and examine the differences between gender and urban-rural areas.

**Results:**

The prevalence of limitations in ADL and physical examination utilization rate among older adults receiving informal care in Shandong Province were 14.12% and 72.31%, respectively. After adjusting for confounders, ADL limitations were negatively correlated with the utilization of physical examination services among older adults receiving informal care (OR = 0.74, 95% CI: 0.64, 0.87, *P* < 0.001), and there were gender and rural-urban differences. The association between ADL limitations and the use of physical examination was statistically significant in older women receiving informal care (OR = 0.65, 95% CI: 0.53, 0.80, *P* < 0.001). And only among urban older adults receiving informal care, those with ADL limitations had lower utilization of physical examination services than participants without ADL limitations (OR = 0.59, 95% CI: 0.47, 0.74, *P* < 0.001).

**Conclusions:**

Our study suggested that the relationship between ADL limitations and the use of physical examination among older adults receiving informal care differed by gender and urban-rural areas in Shandong, China. These findings implied that the government should provide more health resources and personalized physical examination service programs, especially to meet the differential needs of women and urban old adults receiving informal care, to contribute to the implementation of healthy aging strategies.

**Supplementary Information:**

The online version contains supplementary material available at 10.1186/s12877-024-04673-3.

## Introduction

Population aging is one of the major global public health issues. The number of people aged 60 years and above is predicted to exceed 2 billion by 2050 worldwide [1]. According to the data released by China’s National Bureau of Statistics in 2020, older adults are expected to account for 33% of the total population by 2050 in China [2]. Regular physical examination is an important preventive health care service for disease prevention and health promotion. It is considered as one of the most effective measures to alleviate many of the problems related to aging [3, 4]. Moreover, physical examination is widely recognized as the most cost-effective service that can identify diseases or symptoms in the early stages of older adults [3, 5], reducing the socioeconomic burden on patients and communities [6]. Empirical evidence from previous studies suggested that investment in physical examination services could reduce treatment costs and premature mortality significantly [5], and improve quality of life [7]. Therefore, further exploration of the influencing factors of physical examination is essential for disease prevention, which is a high priority to achieve healthy aging.

The decline in physical function is common among older adults [8]. The ability to perform activities of daily living (ADL) is a crucial indicator of physical function in older people [9]. ADL limitations have many negative effects on older adults and their caregivers, including increased healthcare costs, aggravated financial burdens, and decreased quality of life [10, 11]. Informal care provided by family, neighbors, and friends has been the primary source of long-term care for older adults with ADL limitations [12, 13]. On the one hand, influenced by Confucius’ value and the concept of filial piety, family members are the primary care providers for older adults with ADL limitations in China [14]. On the other hand, because China’s formal care system is still in its early stages, there are problems such as insufficient supply, low service quality, and lack of professional service teams, making it practically difficult for many older adults with ADL limitations to access these formal care services [15]. To some extent, informal care improves the quality of life in older adults [16]. However, due to the lack of specialized care services and timely disease diagnosis, older adults with ADL limitations who received informal care may be at increased risk for health problems such as decreased physical functioning, chronic diseases, and depression [17–19], as well as a greater need to prevent diseases and improve health status through physical examination [20]. Several previous studies found an association between ADL limitations and the use of physical examination services [20–23]. Older adults with ADL limitations tended to have fewer physical examinations than those without dysfunction [20, 22]. Nevertheless, most available studies have focused on the factors influencing physical examination [20, 22, 23], and data are out-of-date and may not be representative of the current generation of older adults [20, 21]. There is still a lack of direct emerging research investigating the relationship between ADL limitations and physical examination use. What’s more, most previous studies have focused on the entire older population [20–22], but it is unclear whether there is a correlation between ADL limitations and physical examination among older adults receiving informal care. Exploring the above-mentioned relationship may be beneficial in reducing disease risk and controlling disease progression, which is especially important to prevent disease and contribute to healthy aging [22].

Previous studies have found that older women, due to their lower socioeconomic status and fewer financial resources, are more likely to rely exclusively on informal care and to neglect their health care service needs, including physical examination, in order to reduce household burdens [24]. Gender difference in ADL limitations is also common in older adults. Several cohort studies in the United States have proved that older females had a higher risk of ADL limitations than their male peers [21, 25]. Furthermore, there are potential disparities in health care resources and socioeconomic status between rural and urban areas [26]. It is reasonable to assume that these disparities influence lifestyles, health status, and health care utilization among older adults [27, 28]. Prior research indicated that rural older adults have lower rates of physical examination use [29, 30] and a higher prevalence of ADL limitations than in urban older adults [27]. Although several studies have demonstrated gender and urban-rural disparities in the use of physical examination and ADL limitations separately, it is unclear whether there are gender and urban-rural gaps in the association between ADL limitations and the use of physical examination.

The present study aims to explore the relationship between ADL limitations and the use of physical examination among older adults receiving informal care, as well as gender and rural-urban differences in this association. Based on previous studies, we hypothesized that: (1) older adults receiving informal care with ADL limitations have lower utilization rates of physical examination than their peers without ADL limitations. (2) The relationship between ADL limitations and the use of physical examination varied by gender. Older women with ADL limitations who received informal care have lower utilization of physical examination services. (3) The relationship between ADL limitations and the use of physical examination varied by urban-rural areas. Compared with urban older adults without ADL limitations, the utilization of physical examination services for rural older people with ADL limitations are lower.

## Method

### Study design and participants

The data in this study were obtained from the sixth Health Service Survey of Shandong Province, which was part of the National Health Service Survey (NHSS), conducted in 2018. NHSS is a nationally representative survey organized and directed by the National Health Commission every 5 years since 1993 to fully understand the health status and health service needs of Chinese residents [31]. Shandong province, located in eastern China, is the second-most populated province in China. In 2020, Shandong province’s population aged 60 and over accounted for 20.90% of the total population in China, which is the province with the largest older population [2]. To achieve the representation of the entire population, the sixth Health Service Survey selected subjects using a multi-stage stratified cluster sampling method. First, a total of 20 counties were randomly selected from 137 counties in the eastern, central, and western regions of Shandong Province. Second, five townships were randomly selected from each county, and 100 townships were included in final. Third, two villages (communities) were randomly selected from each township, recruiting a total of 200 villages. Fourth, 60 or more households were surveyed in each village, with 35,264 individuals in 12,938 households completing this survey.

After obtaining informed consent from the respondents, well-trained investigators conducted face-to-face interviews with respondents using a structured questionnaire. Given our focus on the older adult sample, we included participants aged 60 or older in the current study. Besides, a total of 261 dementia respondents and 237 respondents who did not receive informal care from a total of 8,903 older adults interviewed were excluded. After data cleansing (i.e., excluding participants without information of household income, family members, and self-rated health), 8,358 older adults receiving informal care were included in the study. Receiving informal care means that caregiving services were provided by spouses, children and other relatives, neighbors and friends. Figure S1 shows a flowchart of this study sample.

### Measures

#### Physical examination

Physical examination was measured by the question, “Did you have any regular physical examinations in the past 12 months? Tests done due to illness were not included”. The answer options were “yes” (scored as 1) and “no” (scored as 0).

#### Activities of daily living

Participants were asked to answer the question about whether they have difficulty in activities of daily living (ADL). The ADL limitations were measured by Katz Index Scale, which included 6 items: dressing, feeding, bathing, getting in or out of bed, toileting, and bladder and bowel control [32]. Each item was rated on a 4-point scale with the following options: “1 = without difficulty”, “2 = with difficulty but still can complete independently”, “3 = with difficulty and need help” and “4 = unable to complete”. If the answer to any of the items was “with difficulty and need help” or “unable to complete”, they were categorized as older adults with ADL limitations. Finally, ADL limitations were coded as a binomial variable (“yes” or “no”) [33]. The option of “yes” indicated an ADL limitation and “no” manifested no ADL dysfunction. The Chinese version scale, which was used to assess the functional limitations of participants, has been shown to have good validity and reliability [34].

#### Covariates

In this study, covariates included sociodemographic variables, healthy behavior, and health status variables. (1) Sociodemographic variables: age (65–74 years or 75 years and above), gender (male or female), marital status [single (unmarried, divorced, widowed), married]. (2) Economic status variables: the highest education level (illiteracy, primary school, junior school, middle school or above), region (urban, rural), and household income [33] (four types based on percentile, Q1, Q2, Q3 and Q4, from poorest to richest). (3) Health status variables: chronic health conditions [no chronic condition, one chronic condition, multimorbidity (at least two chronic diseases coexist)], and self-rated health (range: 0-100), body mass index [underweight (< 18.5), normal (18.5–24.0), overweight (24.0–28.0), obesity (≥ 28.0)] [35]. Body mass index (BMI) was calculated from weight in kilograms divided by height in square meters.

### Statistical analyses

All statistical analyses were performed using Stata 15.1 (Stata Corp, College Station, TX, USA). The statistical significance was set as a two-tailed and *p*-values less than 0.05. Firstly, descriptive statistics were used to describe characteristics of participants with frequency (percentage) or mean (standard deviation). Student’s t-test for continuous variables and chi-square test for categorical variables were used to examine the differences between older adults who received informal care with and without physical examination. Secondly, we used logistic regression models to assess the association between ADL limitations and the use of physical examination among older adults receiving informal care. In model 1 of the regression analysis, none covariates were included. Model 2 was based on model 1, with additional adjusting for all confounding variables. To explore whether there were disparities in the ADL limitations-physical examination relationship across different gender and region, the interaction terms (ADL limitations× gender and ADL limitations × region) were incorporated into model 3 and 4 of the regression analysis in turn. We also performed the Hosmer-Lemeshow goodness-of-fit test for the four logistic models mentioned above. The test results showed that the four logistic models fit well (Model1-4: *P* = 1.000; *P* = 0.854; *P* = 0.837; *P* = 0.999, respectively). Thirdly, associations between ADL limitations and the use of physical examination stratified by gender and region also were assessed by logistic regression models. The reported confidence intervals (CIs) were calculated at the 95% level.

## Results

### Characteristics of participants

Table 1 shows the participants’ characteristics. A total of 8,358 older adults receiving informal care participated in this study. Among all participants, 81.54% were older adults aged 60–74; 51.9% were female; 63.5% received a low level of education (primary school and below); 84.3% were married; and 51.6% lived in rural areas. Of all respondents, 6,044 have undergone physical examination in the past year. Compared with older adults receiving informal care who did not use physical examination (27.7%), those who did (72.3%) tended to be older, had a lower proportion of males, had less education, and were more likely to live in rural areas. The incidence of limitations in ADL among older adults receiving informal care was 14.1%. More details of descriptive characteristics are provided in Table 1.

**Table 1:** Basic characteristic of the participants among older adults with informal care

Characteristic	N (%)	Physicalexamination		***P***-value ^c^
		Yes (%)	No (%)	
Observations	8358	6044(72.31)	2314(27.69)	
Age (years)				**<0.001**
60-74	6815 (81.54)	4823 (79.80)	1992 (86.08)	
≥75	1543 (18.46)	1221 (20.20)	322 (13.92)	
Gender				**0.001**
Male	4020 (48.10)	2840 (46.99)	1180 (50.99)	
Female	4338 (51.90)	3204 (53.01)	1134 (49.01)	
Region				0.756
Urban	4043 (48.37)	2930 (48.48)	1113 (48.10)	
Rural	4315 (51.63)	3114 (51.52)	1201 (51.90)	
Education				**<0.001**
Illiterate	2576 (30.82)	1946 (32.20)	630 (27.23)	
Primary school	2734 (32.71)	2008 (33.22)	726 (31.37)	
Middle school	1957 (23.41)	1323 (21.89)	634 (27.40)	
High school and above	1091 (13.05)	767 (12.69)	324 (14.00)	
Marital status				0.107
Single ^a^	1311 (15.69)	972 (16.08)	339 (14.65)	
Married	7047 (84.31)	5072 (83.92)	1975 (85.35)	
Household income ^b^				**<0.001**
Q1	2112 (25.27)	1639 (27.12)	473 (20.44)	
Q2	2471 (29.56)	1718 (28.42)	753 (32.54)	
Q3	2060 (24.65)	1444 (23.89)	616 (26.62)	
Q4	1715 (20.52)	1243 (20.57)	472 (20.40)	
BMI				0.909
Underweight	479 (5.73)	343 (5.68)	136 (5.88)	
Normal	3732 (44.65)	2702 (44.71)	1030 (44.51)	
Overweight	3030 (36.25)	2183 (36.12)	847 (36.60)	
Obesity	1117 (13.36)	816 (13.50)	301 (13.01)	
Self-rated health, mean (*SD*)	72.40 (17.86)	72.22 (17.56)	72.87 (18.62)	0.141
Chronic health conditions				**<0.001**
No chronic condition	3713 (44.42)	2485 (41.12)	1228 (53.07)	
One chronic condition	2920 (34.94)	2193 (36.28)	727 (31.42)	
Multimorbidity	1725 (20.64)	1366 (22.60)	359 (15.51)	
Activities of daily living limitations				0.350
Yes	1180 (14.12)	840 (13.90)	340 (14.69)	
No	7178 (85.88)	5204 (86.10)	1974 (85.31)	

Note:

^a^ Singles include those who are unmarried (56, 0.67%), divorced (35, 0.42%) and widowed (1205, 14.42%);

^b^ Quartile 1 (Q1) was the poorest and Quartile 4 (Q4) was the richest;

^c^ The statistical significance was set as a two-tailed and p-values less than 0.05; Student’s t-test for continuous variables and chi-square test for categorical variables were used to examine the differences between older adults who received informal care with and without physical examination;

The total percentage may not equal to 100 due to rounding;

Abbreviations: SD= Standard deviation; BMI= Body mass index

### Association between ADL limitations and the use of physical examination

Table 2 presents the relationship between the different ADL and the use of physical examination among older adults receiving informal care in Shandong, China. In Model 1, there was no statistical significance between ADL limitations and utilization of physical examination services (OR = 0.94, 95% CI: 0.82, 1.07). When control variables were adjusted for Model 2, older adults with ADL limitations had lower utilization of physical examination services than their peers without ADL limitations (OR = 0.74, 95% CI: 0.64, 0.87).

**Table 2:** Association between ADL limitations and physical examination among older adults with informal care

Characteristics	Model 1^a^		Model 2^b^		Model 3^c^		Model 4^d^	
	OR (95% CI)	***P***-value	OR (95% CI)	***P***-value	OR (95% CI)	***P***-value	OR (95% CI)	***P***-value
*Main terms*								
ADL limitations (No ^Ref^)								
Yes	0.94 (0.82, 1.07)	0.350	0.74 (0.64, 0.87)	**<0.001**	0.86 (0.70, 1.07)	0.172	0.55 (0.44, 0.68)	**<0.001**
Gender (Male ^Ref^)								
Female			1.07 (0.96, 1.19)	0.215	1.11 (0.99, 1.25)	0.066	1.07 (0.96, 1.19)	0.225
Region (Urban ^Ref^)								
Rural			0.94 (0.84, 1.04)	0.213	0.94 (0.85, 1.04)	0.226	0.87 (0.78, 0.97)	**0.012**
*Interaction term*								
ADL limitations×Gender (Without ADL limitations×Male ^Ref^ )								
ADL limitations×Female					0.76 (0.57, 1.00)	**0.050**		
ADL limitations×Region (Without ADL limitations×Urban ^Ref^ )								
ADL limitations×Rural							1.70 (1.29, 2.25)	**<0.001**
*Controls*								
Age (65–74 ^Ref^)								
≥75			1.51 (1.30, 1.74)	**<0.001**	1.51 (1.31, 1.75)	**<0.001**	1.52 (1.32, 1.76)	**<0.001**
Education (Illiterate ^Ref^)								
Primary school			0.94 (0.82, 1.07)	0.361	0.94 (0.82, 1.07)	0.326	0.94 (0.83, 1.08)	0.389
Middle school			0.73 (0.63, 0.84)	**<0.001**	0.73 (0.63, 0.84)	**<0.001**	0.73 (0.63, 0.84)	**<0.001**
High school and above			0.79 (0.66, 0.95)	**0.011**	0.79 (0.66, 0.95)	**0.011**	1.78 (0.65, 0.94)	**0.010**
Marital status ^e^ (Single ^Ref^)								
Married			1.12 (0.97, 1.29)	0.139	1.11 (0.96, 1.28)	0.158	1.12 (0.97, 1.30)	0.125
Household income ^f^ (Q1 ^Ref^)								
Q2			0.69 (0.60, 0.79)	**<0.001**	0.69 (0.60, 0.80)	**<0.001**	0.70 (0.61, 1.80)	**<0.001**
Q3			0.70 (0.61, 0.81)	**<0.001**	0.70 (0.61, 0.81)	**<0.001**	0.71 (0.61, 0.82)	**<0.001**
Q4			0.78 (0.66, 0.92)	**0.004**	0.78 (0.66, 0.92)	**0.004**	0.78 (0.66, 0.92)	**0.003**
BMI (Underweight ^Ref^)								
Normal			1.12 (0.90, 1.39)	0.297	1.12 (0.90, 1.40)	0.291	1.12 (0.90, 1.40)	0.294
Overweight			1.08 (0.87, 1.35)	0.477	1.09 (0.87, 1.36)	0.459	1.08 (0.87, 1.35)	0.484
Obesity			1.08 (0.84, 1.39)	0.541	1.09 (0.85, 1.39)	0.505	1.08 (0.84, 1.38)	0.549
Self-rated health			1.00 (1.00, 1.01)	**0.013**	1.00 (1.00, 1.01)	**0.012**	1.00 (1.00, 1.01)	**0.010**
Chronic health conditions (No chronic condition ^Ref^)								
One chronic condition			1.54 (1.38, 1.73)	**<0.001**	1.55 (1.38, 1.73)	**<0.001**	1.55 (1.38, 1.73)	**<0.001**
Multimorbidity			2.03 (1.75, 2.35)	**<0.001**	2.03 (1.75, 2.35)	**<0.001**	2.04 (1.76, 2.36)	**<0.001**

Note: ADL= Activities of daily living; CI= Confidence interval; OR= odds ratio

^a^ Model 1: Unadjusted;

^b^ Model 2: Additionally adjusted for gender, region, age, education, marriage status, BMI, household income, self-rated health, and chronic health conditions;

^c^ Model 3: Adjusted for model 2 criteria and the interaction between activities of daily living and gender;

^d^ Model 4: Adjusted for model 2 criteria and the interaction between activities of daily living and region;

^e^ Singles include those who are unmarried (56, 0.67%), divorced (35, 0.42%) and widowed (1205, 14.42%);

^f^ Quartile 1 (Q1) was the poorest and Quartile 4 (Q4) was the richest;

Statistically significant *p* < 0.05 values are indicated in bold

### Effects of gender and region on the association between ADL limitations and the use of physical examination

Model 3 illustrates the interaction between ADL limitations and gender in the prediction of physical examination services with adjusted 95% CI based on model 2. There was significant interaction in ADL limitations × gender with physical examination (OR = 0.76, 95% CI: 0.57, 1.00). In addition, the interaction term between ADL limitations and region was included in Model 4 to further analyze whether the relationship between ADL limitations and utilization of physical examination services varied by urban-rural areas. Compared with the urban older adults without ADL limitations, the utilization rates of physical examination services for the rural older people with ADL limitations are higher (OR = 1.70, 95% CI: 1.29, 2.25).

### Association between ADL limitations and the use of physical examination in subgroups

As shown in Table 3, the relationship between ADL limitations and the use of physical examination stratified by gender is reported. Participants with ADL limitations were associated with lower utilization of physical examination services (OR = 0.65, 95% CI: 0.53, 0.80, *P* < 0.001) only in older women receiving informal care. Table 4 presents the relationship between ADL limitations and the use of physical examination stratified by region. The association between ADL limitations and the use of physical examination was found only in urban older adults receiving informal care. ADL limitations were negatively correlated with the utilization of physical examination services (OR = 0.59, 95% CI: 0.47, 0.74, *P* < 0.001).

**Table 3:** Association between ADL limitations and physical examination stratified by gender

Variable	Male (*n* = 4020)				Female (*n* = 4338)			
	Model 1^a^		Model 2^b^		Model 1^a^		Model 2^b^	
	OR (95% CI)	***P***-value	OR (95% CI)	***P***-value	OR (95% CI)	***P***-value	OR (95% CI)	***P***-value
ADL limitations								
No	1.00 (reference)		1.00 (reference)		1.00 (reference)		1.00 (reference)	
Yes	1.07 (0.88, 1.31)	0.490	0.86 (0.69, 1.07)	0.184	0.82 (0.68, 0.99)	**0.041**	0.65 (0.53, 0.80)	**<0.001**

Note: ADL= Activities of daily living; CI= Confidence interval; OR= odds ratio

^a^ Model 1 was unadjusted;

^b^ Model 2 was adjusted for region, age, education, marriage status, BMI, household income, self-rated health, and chronic health conditions;

Statistically significant *p* < 0.05 values are indicated in bold

**Table 4:** Association between ADL limitations and physical examination stratified by region

Variable	Urban (*n* = 4043)				Rural (*n* = 4315)			
	Model 1^a^		Model 2^b^		Model 1^a^		Model 2^b^	
	**OR (95% CI)**	***P*** **-value**	**OR (95% CI)**	***P*** **-value**	**OR (95% CI)**	***P*** **-value**	**OR (95% CI)**	***P*** **-value**
ADL limitations								
No	1.00 (reference)		1.00 (reference)		1.00 (reference)		1.00 (reference)	
Yes	0.71 (0.58, 0.87)	**0.001**	0.59 (0.47, 0.74)	**<0.001**	1.17 (0.97, 1.40)	0.104	0.88 (0.71, 1.08)	0.208

Note: ADL= Activities of daily living; CI= Confidence interval; OR= odds ratio

^a^ Model 1 was unadjusted;

^b^ Model 2 was adjusted for gender, age, education, marriage status, BMI, household income, self-rated health, and chronic health conditions;

Statistically significant *p* < 0.05 values are indicated in bold

## Discussion

This study aimed to explore the relationship between ADL limitations and the use of physical examination among older adults receiving informal care, and to further analyze the gender and rural-urban differences in this association. Our findings suggested that ADL limitations were associated with lower utilization of physical examination services among older adults receiving informal care in Shandong, China. We also found gender and urban-rural disparities in the relationship between ADL limitations and the use of physical examination. Specifically, ADL limitations were negatively associated with the use of physical examination among older women, and a significant correlation was only found in urban areas.

The current study indicated that ADL limitations were negatively associated with the use of physical examination among older adults receiving informal care in Shandong, China. This finding was consistent with previous studies that ADL limitations were connected with decreased utilization of physical examination services in older adults [20–22]. There are several possible explanations for this finding. First, this could be due to a shortage of qualified primary healthcare providers [11]. Informal caregivers generally lack specialized care knowledge and skills training, and they tend to focus on health care for the “dysfunction” itself, ignoring the importance of using preventive health services, especially for older adults with ADL limitations [36, 37]. Meanwhile, due to limited human resources and heavy healthcare tasks, primary healthcare institutions lack sufficient doctors who can provide preventive care services to patients with ADL limitations [38]. Second, family members are the primary caregivers for older adults with ADL limitations in China [12, 13]. Because family caregivers lack basic care knowledge, the standard of care does not meet the needs of elderly care recipients with ADL imitations [37]. Older adults receiving informal care may be unable to share their social or healthcare needs with family or friends, for fear of being perceived as a burden by their families [39]. This may make it difficult for their physical examination needs to be truly met.

We found a gender disparity in the association between ADL limitations and the use of physical examination. ADL limitations were inversely associated with the use of physical examination only in older women receiving informal care, but not in older men. There is one possible explanation that may account for the gender difference. Women are socialized with an emphasis on home-centered gender roles, especially in China [24]. On the one hand, older women generally believe that men are the center of the family and the most important members [40]. Thus, older women with ADL limitations are more concerned about causing problems for their families and neglecting their need for preventive physical examination services [24, 41]. On the other hand, older women may have more experience with caregiving than older men [42, 43]. If older women have a spouse with ADL limitations, they may be better able to provide care services than husbands who are less able to care for themselves [42, 44]. This makes it easier for older men with ADL limitations to meet their healthcare needs, including the use of physical examination. Additionally, medical costs are considered to be a possible barrier to older women using physical examination services. Previous studies have shown that the role of household financial provider is generally assumed by older men [40]. Older women, with limited financial resources and lower incomes [45], are more likely to be anxious that physical examination would detect health problems and lead to increased expenditures.

Notably, inconsistent with hypothesis 3 of this study, we found that there were urban-rural differences in the association between ADL limitations and the use of physical examination services among older adults receiving informal care, and only a negative association was demonstrated in urban older adults. This finding may be somewhat surprising, given that urban older adults in China have greater financial and medical resources than those in rural areas [26]. One possible reason for this finding is that the infrequent and low-quality care provided by informal caregivers, as well as ADL limitations, increase the risk of illness and financial burden for older adults [10, 17, 18]. Compared to households living in urban areas, rural households have lower financial incomes and limited disposable funds [46]. As a result, rural older adults with ADL limitations may be more active in using physical examination services in order to prevent and control the disease progression and keep it from further deterioration, avoiding a greater financial burden. Another reason may be that China has a dual economy structure, which has created a division in the social and family structure between urban and rural areas [28]. Compared to rural areas where extended families predominate, nuclear families are becoming more common in cities [47]. With the rapid socio-economic development in China, the pressure on today’s young and middle-aged people living in urban areas has increased dramatically [48]. Older adults with ADL limitations living in urban areas may prefer not to undergo physical examination for fear of wasting their children time at work and increasing their burden. Simultaneously, the greater availability of health care services in urban areas [28], and it is convenient to seek medical care and purchase medication when health problems arise. These may, to some extent, weaken the preventive health behavior of urban older adults with ADL limitations. More empirical studies are urgently needed to further validate our findings in the future, as the above analysis only partially explains them.

Our findings provided new practical experience and operational interventions for improving the utilization of physical examination services and disease prevention. First, policymakers should integrate basic preventive health care services into the health insurance system and implement community-based physical examination education to achieve health promotion and disease prevention goals. Second, the government should also optimize physical examination services to meet individualized health needs and reduce barriers to seeking preventive care. In particular, free transportation or home-based physical examination should be considered for older adults receiving informal care who have functional problems. Third, the government should strengthen financial investment in community-based elderly care centers and establish a long-term care system that combines formal and informal care model, especially to meet the health service needs of older adults with ADL limitations. Finally, primary health care should focus on reminding the older people receiving informal care to participate in physical examination promptly, strengthening health management, and achieving early detection and intervention of diseases, especially for older women with ADL limitations and urban older adults with ADL dysfunction.

Several limitations of this study need to be acknowledged when interpreting the results. First, this was a cross-sectional study that may not investigate the temporal relation between ADL limitations and physical examination use. In future studies, a longitudinal design can be used to enrich the exploration of the above relationship. Second, information including economic status and some other variables was self-reported, which may lead to biased recollection of certain information, to occur measurement errors and classification errors. Third, for the data constraints in the sixth Health Service Survey of Shandong Province questionnaire, future studies could collect more information on instrumental activity of daily living (IADL) limitations, for a more comprehensive exploration of functional limitations. Finally, the present study was only applicable to older adults receiving informal care in Shandong Province, China, and other populations need to be verified in future studies.

## Conclusions

This study explores the relationship between ADL limitations and the use of physical examination, as well as investigates whether this relationship varies by gender and urban-rural areas. According to our findings, older adults receiving informal care with ADL limitations had lower utilization rates of physical examination than those without ADL limitations. The association was statistically significant in older women receiving informal care. Only among urban older adults receiving informal care did those with ADL limitations use fewer physicalexamination services than participants without ADL limitations. To better meet the preventive health care needs among older adults with ADL limitations, the government should provide more health resources and incentives, as well as develop personalized physical examination service programs, especially to meet the differential needs of women and the urban older adults receiving informal care, in order to contribute to the implementation of healthy aging strategies.

### Electronic supplementary material

Below is the link to the electronic supplementary material.

Supplementary Material 1

## Data Availability

The datasets used in the current study are not publicly available due to the confidential policy but are available from the corresponding author on reasonable request.

## Abbreviations

ADL: Activities of daily living
NHSS: The National Health Service Survey
HRQOL: Health-related quality of life
BMI: Body mass index
IADL: Instrumental activity of daily living
