# Supplementary figures and images for: Activities of daily living limitations and the use of physical examination among older adults with informal care in China: do gender and residence make differences?
Source: BMC Geriatr. 2024 Jan 23;24:87. doi: 10.1186/s12877-024-04673-3 (PMC10807140; doi:10.1186/s12877-024-04673-3)

**Supplementary material**


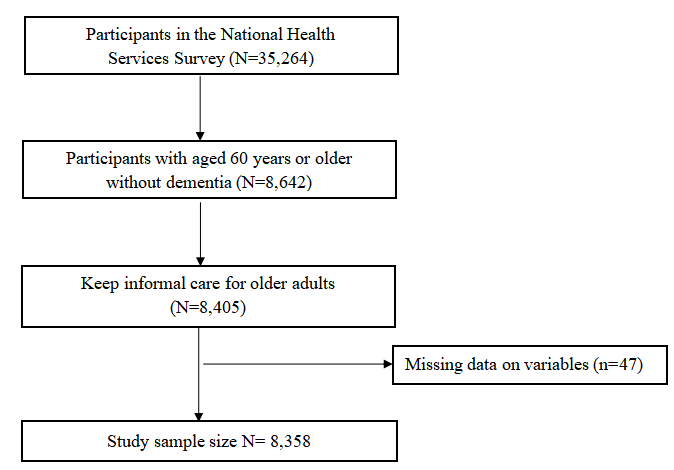


**Figure S1 Flow of participants into study sample.**

Supplement: Supplementary file 1 — Supplementary Material 1 [file 12877_2024_4673_MOESM1_ESM.doc]
